# Macrophages in Zebrafish Models of Liver Diseases

**DOI:** 10.3389/fimmu.2019.02840

**Published:** 2019-12-04

**Authors:** Arkadi Shwartz, Wolfram Goessling, Chunyue Yin

**Affiliations:** ^1^Division of Genetics, Brigham and Women's Hospital, Harvard Medical School, Boston, MA, United States; ^2^Division of Genetics, Brigham and Women's Hospital, Harvard Medical School, Boston, MA, United States; ^3^Harvard Stem Cell Institute, Cambridge, MA, United States; ^4^Dana-Farber Cancer Institute, Harvard Medical School, Boston, MA, United States; ^5^Broad Institute, Massachusetts Institute of Technology and Harvard, Cambridge, MA, United States; ^6^Division of Health Sciences and Technology, Harvard and Massachusetts Institute of Technology, Boston, MA, United States; ^7^Division of Gastroenterology, Massachusetts General Hospital, Harvard Medical School, Boston, MA, United States; ^8^Division of Gastroenterology, Hepatology and Nutrition and Division of Developmental Biology, Cincinnati Children's Hospital Medical Center, Cincinnati, OH, United States

**Keywords:** kupffer cells, monocytes, regeneration, NAFLD, ALD, hepatocellular carcinoma

## Abstract

Hepatic macrophages are key components of the liver immunity and consist of two main populations. Liver resident macrophages, known as Kupffer cells in mammals, are crucial for maintaining normal liver homeostasis. Upon injury, they become activated to release proinflammatory cytokines and chemokines and recruit a large population of inflammatory monocyte-derived macrophages to the liver. During the progression of liver diseases, macrophages are highly plastic and have opposing functions depending on the signaling cues that they receive from the microenvironment. A comprehensive understanding of liver macrophages is essential for developing therapeutic interventions that target these cells in acute and chronic liver diseases. Mouse studies have provided the bulk of our current knowledge of liver macrophages. The emergence of various liver disease models and availability of transgenic tools to visualize and manipulate macrophages have made the teleost zebrafish (*Danio rerio*) an attractive new vertebrate model to study liver macrophages. In this review, we summarize the origin and behaviors of macrophages in healthy and injured livers in zebrafish. We highlight the roles of macrophages in zebrafish models of alcoholic and non-alcoholic liver diseases, hepatocellular carcinoma, and liver regeneration, and how they compare with the roles that have been described in mammals. We also discuss the advantages and challenges of using zebrafish to study liver macrophages.

## Introduction

The liver is the largest internal organ in the body and exerts vital metabolic and immunological functions. Liver disease is a major health burden and accounts for ~2 million deaths per year worldwide (1). Liver transplantation is often the only curative option for patients with liver failure due to acute or chronic liver injury, and thus there is an urgent unmet need for alternative treatment.

The liver contains the largest number of tissue-resident macrophages that account for 80–90% of all macrophages in the body (2). These so-called Kupffer cells are considered to be self-renewing and non-migratory. During homeostasis, they exert phagocytic function to clear pathogens that reach the liver through the circulating blood. This macrophage population also maintains immunological tolerance in the liver to reduce accidental immune responses. During injury, Kupffer cells become activated and secrete pro-inflammatory cytokines and chemokines to recruit bone marrow-derived monocytes to the liver (3). Extensive research in samples from patients with liver diseases and rodent models of liver injury has revealed that both Kupffer cells and monocyte-derived liver macrophages play critical roles in hepatic steatosis, inflammation, fibrosis, and cancer, making them appealing therapeutic targets. Developing macrophage-based therapy, however, is challenging because it is a highly heterogeneous population. In fact, a recent study using single-cell RNAseq has identified 10 subpopulations of macrophages in human control and cirrhotic livers (4). Furthermore, macrophages are very plastic and often have multiple and sometime opposing functions in promoting liver disease progression vs. repairing injured liver (5).

The teleost zebrafish, an increasingly popular vertebrate model for studying development and genetics, has shown promise in bringing new insights into our understanding of the ontogeny of liver macrophages and their responses to injury. Zebrafish form a functional liver by just 4 days post fertilization (6). Despite some architectural differences, the zebrafish liver contains a highly similar parenchymal and non-parenchymal cell inventory as the mammalian liver. Taking advantages of the transparent larva and the accessibility to genetic manipulation, researchers have generated transgenic fluorescent reporter strains to mark individual liver cell types, enabling real-time tracking of their morphology and behaviors during development and injury (6). Zebrafish have been used in translational research modeling various liver diseases such as drug-induced acute liver failure, cholestasis, non-alcoholic liver disease, alcoholic liver disease, and cancer (6–8). These studies have demonstrated that the signaling pathways governing liver injury responses are highly conserved between zebrafish and mammals. Zebrafish are also an excellent *in vivo* model system for studying the innate immune system. The embryos have functional macrophages at 1 day post fertilization and neutrophils by 2 days (9). The zebrafish macrophages have conserved marker gene expression and functions as their mammalian counterparts. They can be easily visualized during homeostasis and inflammatory processes using the fluorescent reporter lines (9). Table 1 summarizes the tools for observing and manipulating macrophages in zebrafish.

**Table 1:** Tools to study macrophages in Zebrafish.

**Markers for macrophages**		
**Dye**		
Neutral Red	Marks live macrophages	(10)
**Riboprobes for** ***in situ*** **hybridization**		
*csf1ra*	Also labels neural crests	(11)
*mfap4*		(12)
*cxcr3.2*		(12)
*mpeg1*		(12, 13)
*mpeg1.2*		(14)
*ptpn6*		(12)
***Antibody***		
L-plastin	Pan-leukocyte marker	(15, 16)
Mpeg		(15)
WCL15	Antigen unknown	(11, 17, 18)
**Transgenic Reporter Line**		
*Tg(mpeg1:GFP);* *Tg(mpeg1:mCherry);* *Tg(mpeg1:Gal4-VP16)*		(13)
*Tg(mpeg1:Dendra2)*	Photoconvertible protein	(19)
*Tg(mpeg1:Kaede)*	Photoconvertible protein	(20)
*Tg(mpeg1:Cre)*	Applications include lineage tracing and tracking macrophage-dependent cytoplasmic transfer.	(21)
*TgBAC(csf1ra:Gal4-VP16)* *TgBAC(csf1ra:EGFP)*	Marks mononuclear phagocytes	(22) (23)
*Tg(mfap4:quoise);* *Tg(mfap4:GFP)*		(24, 25)
*Tg(tnfα:EGFP-F)*	Marks activated macrophage	(26)
*Tg(irg1:EGFP)*	Marks activated macrophage	(27)
*TG(CORONIN1A:GFP)*	Marks myeloid cells and lymphocytes	(28)
**MACROPHAGE-SPECIFIC ABLATION MODELS**		
**Chemicals**		
Clodronate liposomes		(29, 30)
Carrageenan		(31)
**Macrophage-deficient mutants and morphants (morpholino-injected animals)**		
*Panther/csfr1a* mutant	Reduced primitive macrophages	(11, 20, 32)
*irf8* mutant	Reduced macrophages and increased neutrophils	(33, 34)
*irf8* morphant	Reduced macrophages and increased neutrophils	(33)
*Pu.1* morphant	Lacks macrophage up to 3 days post fertilization; shows mortality after day 7.	(35, 36)
**Nitroreductase-based macrophage ablation**		
*Tg(mpeg1:NTR-eYFP)*		(37)
*Tg(mpeg1:Gal4-VP16;* *UAS:NTR-mCherry);*		(38)
**MODELS WITH IMPAIRMENT IN MACROPHAGE**		
**MIGRATION/CHEMOTAXIS**		
*cxcr3.2* mutant		(39)
*cxcr3.2* morphant		(12)
Thymosin β4 sulfoxide treatment		(40)

**Figure 1:**
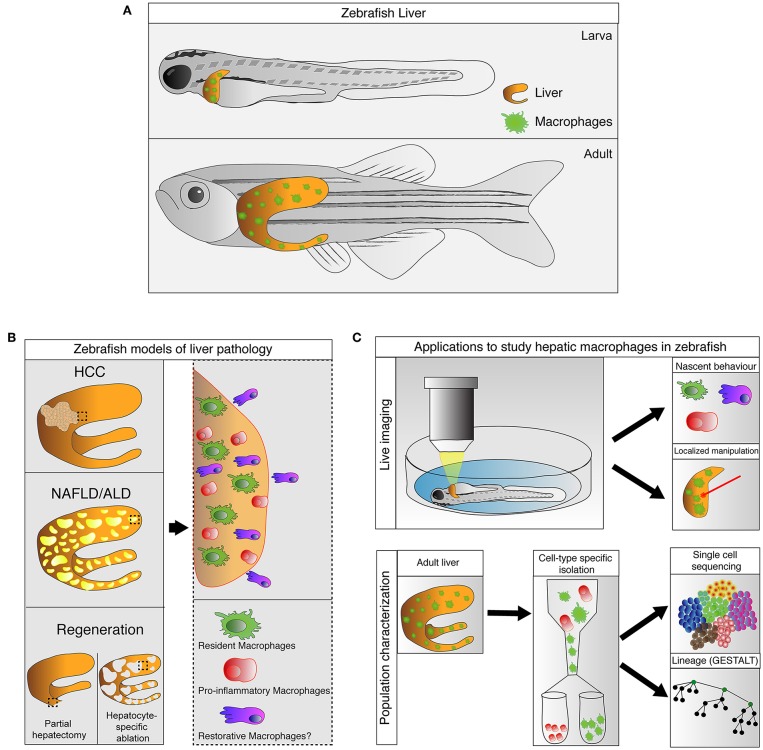
Zebrafish, an emerging model for study hepatic macrophages. **(A)** Hepatic macrophages are present in the zebrafish liver at both larval and adult stages. **(B)** Increases in macrophage numbers have been observed in zebrafish models of liver pathology include non-alcoholic liver disease (NAFLD), alcoholic liver disease (ALD), and hepatocellular carcinoma (HCC), as well as in liver regeneration after partial hepatectomy and hepatocyte-specific ablation (left). Involvement of heterogeneous macrophage populations has been implicated in these models (right). **(C)** Current and potential applications available in zebrafish to study hepatic macrophages. Zebrafish larva is accessible for live imaging, allowing characterization of macrophage behaviors during early stages of immune responses. The live imaging platform in larva can also be utilized for laser-mediated localized manipulations of gene expression and cell ablation. Technologies such as GESTALT (genome editing of synthetic target arrays for lineage tracing) and single cell RNA-sequencing can be utilized to study the ontology and plasticity of macrophages in healthy and injured livers at a population level.

Recent studies have confirmed the presence of macrophages in the livers of larval and adult zebrafish in physiological and pathological conditions. In this review, we provide an overview of the origin and development of hepatic macrophages in zebrafish. We highlight the recent advances where zebrafish transgenesis and imaging approaches reveal new aspects of macrophage functions in liver diseases. In particular, we focus on their roles in non-alcoholic and alcoholic liver disease, hepatocellular carcinoma, and liver regeneration. The capabilities and potential of the zebrafish model in studying liver macrophages are also discussed (summarized in Figure 1).

## The Origin of Hepatic Macrophages in Zebrafish

### Overview of Zebrafish Hematopoiesis

Similar to mammals, the development of the zebrafish hematopoietic system is characterized by several distinct waves (41, 42). The first wave, referred as primitive, occurs during early somitogenesis in the ventral lateral mesoderm and rostral blood island (RBI) at ~11 h post fertilization (hpf). The progenitors converge to the midline to form the intermediate cell mass, which is the primary site for primitive hematopoiesis and functionally equivalent to mammalian yolk sac blood islands. The process continues at ~24 hpf in the RBI during which the transient erythro-myeloid precursors (EMPs) are formed. The EMPs have limited lineage differentiation potential and lack the self-renewal capacity (43). The second or definitive wave of hematopoiesis starts at ~36 hpf when the first hematopoietic stem cells (HSCs) emerge from the ventral wall of the dorsal aorta (VDA) in the aorta-gonad mesonephros (AGM) region. This process is conserved among vertebrate species and gives rise to a multipotent cell type that can contribute to the entire hematopoietic lineage (44, 45). Another conserved feature between mammals and zebrafish is the migratory ability of the HSCs as they seed in different anatomical niches in order to differentiate and proliferate. Subsequently, hematopoiesis proceeds in the distal region of the tail, which is known as the caudal hematopoietic tissue (CHT) and represents the equivalent of the mammalian fetal liver (46). At about 96 hpf, the HSCs migrate either from the CHT or directly from the AGM to colonize the pronephros (47). There they will constitute the kidney, which corresponds to the mammalian bone marrow, to provide the adult zebrafish with hematopoiesis throughout their lifespan.

### Tissue Resident Macrophages Arise From the HSC Origin, a Lesson From Fish

The zebrafish innate immune system is mainly composed of macrophages and neutrophils. Both are derived from the myeloid lineage that emerges during the primitive hematopoietic wave from the cells in the lateral plate mesoderm expressing *Spi-1 proto-oncogene* (*spi1*) and *lymphocyte cytosolic protein 1 (lcp1)* also known as *L-plastin* (48, 49). Definitive hematopoiesis continues to contribute to the myeloid lineage and sustains its functionality throughout the lifespan. The innate immune system solely provides zebrafish with immune defense during the first month of life until the adaptive immune system fully develops (50).

The macrophage population consists of tissue-resident macrophages, bone marrow-derived recruited macrophages, and peritoneal macrophages. Resident macrophages are present in most tissues across the body and fulfill vital functions in homeostasis (51). It has been shown that the early EMPs populate different organs during development to form most of the resident macrophages in mice. This macrophage population acquires specialized, tissue-resident properties, and harbors self-renewing potential to maintain the adult population (52–55). One exception is the gut, where the macrophage population is continually replenished by circulating monocytes that differentiate into the mature resident macrophages (56).

Studies of resident macrophages in zebrafish have provided novel assessments of their origin. Recent work identified the age-dependent origin of microglia (57). While the primitive macrophages give rise to a transient population of microglia during the early larval stage, the adult microglia originate from the *cmyb*-dependent HSCs. Similar observations were made in adult zebrafish Langerhans cells and several other resident-macrophage populations (58), challenging the current model of the erythro-myeloid origin of tissue macrophages. The zebrafish results are supported by a recent mammalian study (59), although there is still much controversy in the field (54).

### Origin of Liver Resident Macrophages in Zebrafish

The liver is continuously exposed to antigens, microbial products, and xenobiotics. To adapt to such an environment, the liver harbors the largest population of macrophages among the solid organs and is constantly patrolled by circulating monocytes. Based on the ontogeny studies conducted mainly in mice, Kupffer cells originate from the yolk sac-derived erythro-myeloid progenitors that express macrophage colony stimulating factor 1 receptor (CSF1R) and are self-renewing (54). Recently, HSCs and some common circulating precursors have also been implicated in the development of Kupffer cells (53, 59, 60).

Resident macrophages have been observed in the adult zebrafish liver (58, 61–63). In elegant work, He and colleague utilized a laser-mediated temporal-spatially resolved cell labeling IR-LEGO-CreER-loxP system to mark cells within different hematopoietic compartments during distinctive waves of hematopoiesis and trace the destination of the labeled cells in adults (58). Followed by fine fate-mapping analysis, they showed that most of the primary tissue-resident macrophages in adult zebrafish, including those in the liver, are derived from the VDA, suggesting their HSC origin. This work illustrates how zebrafish can offer unique tools to elucidate the ontogeny of hepatic macrophages, which is a challenging topic in hepatology.

## Macrophages in Zebrafish Models of Liver Diseases

### Non-alcoholic and Alcoholic Liver Disease

Non-alcoholic fatty liver disease (NAFLD) and alcoholic liver disease (ALD) are among the leading causes of liver-related morbidity and mortality and primary indications for liver transplantation. In both diseases, extensive hepatic lipid accumulation caused by metabolic stress or alcohol consumption induces hepatocyte cell death (64). Damaged hepatocytes release danger-associated molecular patterns (DAMPs) to trigger activation of Kupffer cells and infiltration of circulating monocytes (3). Macrophages play divergent roles in NAFLD and ALD: they exhibit a pro-inflammatory phenotype during disease progression (65, 66) and become anti-inflammatory and tissue-protective during disease regression (66, 67). Feeding zebrafish larvae with a high cholesterol diet (5% cholesterol w/w) for a week can cause elevated triglyceride and total cholesterol levels and lipid accumulation in the body. The animals develop macrovesicular steatosis in the liver by 1-week of feeding and display ballooning degeneration by 3 weeks (68). De Oliveira et al. showed that short-term feeding with a high fat diet (HFD) results in clustering of macrophages in the zebrafish larval liver (61). Whereas, the macrophages in the control livers constantly patrol the environment, the macrophages in the HFD-fed liver are more stationary and adopt a rounder morphology. They start to express TNFα, a consensus marker of M1 macrophages, consistent with activation and polarization of these cells (26).

It has been reported by multiple groups that acute and chronic ethanol treatment can induce hepatic steatosis in larval and adult zebrafish, respectively (69–73). In human and mouse, after alcohol consumption, ethanol enters the blood circulation through the gastrointestinal tract and reaches the liver via the portal vein (74). Ethanol is metabolized in the liver mainly by alcohol dehydrogenase ADH1 and cytochrome P450 2E1/CYP2E1 enzymes. Zebrafish have analogs of ADH1 and CYP2E1 that are capable of metabolizing ethanol (75). Treatment with pharmacological inhibitors of ADH1 and CYP2E1 blocks ethanol-induced hepatic steatosis in zebrafish larvae, indicating that steatosis is caused by ethanol metabolism (76). Zebrafish alcoholic injury models are achieved by aqueous exposure of the animals to ethanol. Thus, ethanol exposure can go through multiple routes, including the gastrointestinal tract, gill, and skin. Since the expression of ethanol-metabolizing enzymes has not been characterized at the tissue level in zebrafish, it is not clear whether tissues other than the liver participate in ethanol metabolism.

**Figure 2:**
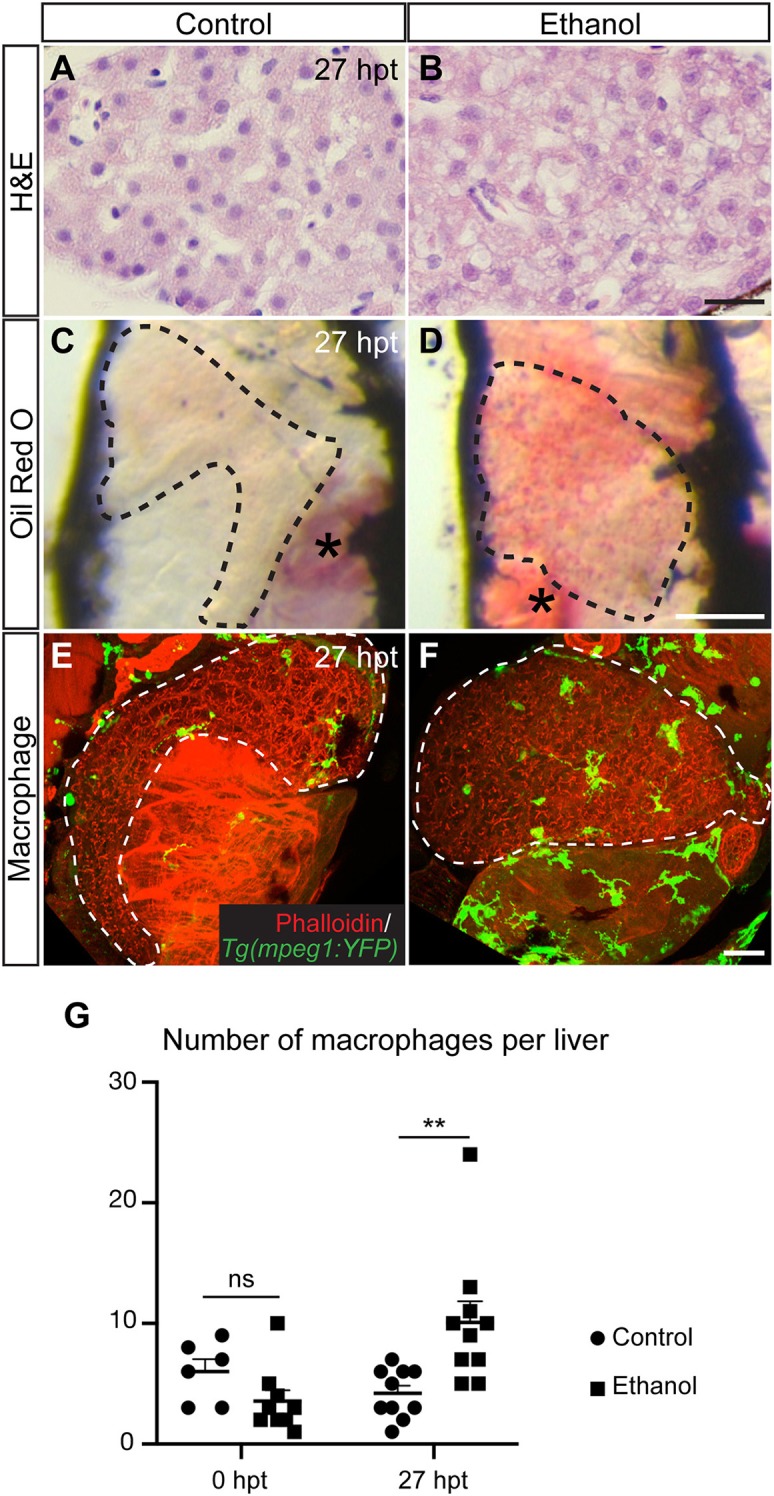
Acute ethanol treatment causes hepatic steatosis and increases macrophage numbers in larval zebrafish. **(A,B)** Hematoxylin and eosin (H&E) staining of the paraffin sections showing the livers in a control larva **(A)** and a larva treated with 2% ethanol from 96 to 120 h post fertilization **(B)**. The livers were harvested at 27 h post treatment (hpt). Scale bar, 20 μm. **(C,D)** Representative images of the whole-mount Oil Red O staining in the control **(C)** and ethanol-treated larvae **(D)**. Dashed line outlines the liver. Lateral view, anterior is to the top. Oil Red O also stains the swim bladder (asterisk in C) and the residual yolk tissue (asterisk in D). Scale bar, 250 μm. **(E,F)** Confocal three-dimensional projections showing *Tg(mpeg1:YFP)*-expressing macrophages (green) in the whole liver at 27 hpt. Phalloidin staining (red) that labels cell cortex is used for recognizing various organs. Ventral views, anterior is to the top. Dashed line outlines the liver. Scale bar, 30 μm. **(G)** Numbers (mean±s.e.m.) of macrophages per liver at 0 hpt (left) and 27 hpt (right). Statistical significance was calculated by one-way ANOVA and Tukey's *post-hoc* test. ^**^p < 0.01, ns, not significant. This figure is reproduced with permission from Zhang et al. (73) and *Disease Models & Mechanisms*.

In the acute alcoholic liver injury model, exposing 4-day-old zebrafish larvae to 2% ethanol for 24 h causes hepatic steatosis (Figure 2) (71, 76). At this stage, the yolk provides the animal all the nutrients and is likely the source of fat in steatosis (76). Mammalian studies indicate that alcohol exposure increases the ratio of reduced nicotinamide adenine dinucleotide/oxidized nicotinamide adenine dinucleotide and subsequently impairs mitochondrial β-oxidation of fatty acids (77). Alcohol exposure also promotes lipogenesis and inhibits fatty acid oxidation by regulating the transcription factors of lipid metabolism. In the zebrafish acute alcoholic liver injury model, alcohol-induced lipogenesis requires activation of the sterol regulatory element binding protein (SREBP) transcription factors and involves the unfolded protein response pathway (71, 76, 78). In zebrafish larvae, acute ethanol exposure also prompts hepatic stellate cells to express extracellular matrix proteins and causes dilatation of the hepatic blood vessels (73). One day after ethanol is removed, there is an increase in the number of macrophages in the treated liver (Figure 2), accompanied with increased hepatic angiogenesis and hepatic stellate cell proliferation (73). In mammalian models of chronic liver injury, macrophages are the source of vascular endothelial growth factor that promotes angiogenesis (79). They also have dual function in fibrosis: both Kupffer cells and monocyte-derived macrophages are profibrogenic during fibrosis progression as they secrete TGFβ1 and PDGF to activate hepatic stellate cells and mediate the survival of myofibroblasts (80). When the insults are removed, monocyte-derived macrophages become antifibrotic to aid in the resolution of fibrosis. In our opinion, the zebrafish acute alcoholic liver injury model is useful for studying the initial responses of macrophages, endothelial cells, and hepatic stellate cells upon the addition and removal of ethanol. Such responses trigger the subsequent cascades of events underlying disease progression and regression.

It is important to note that NAFLD and ALD are chronic diseases progressing from hepatic steatosis to steatohepatitis, and further to fibrosis and cirrhosis, increasing the risk for hepatocellular carcinoma. The zebrafish NAFLD and ALD models described above are mainly based on short-term treatment at larval stages and do not recapitulate the full spectrum of the disorders in human. Fibrosis has not been observed in the larval NAFLD and ALD models, which could be due to the fact that the zebrafish liver does not have the portal-central arrangement as the mammalian liver. The duration of the experiments may not be long enough for fibrosis to develop. Therefore, it is important to validate the findings in adult chronic injury models and mammalian systems.

### Hepatocellular Carcinoma

Hepatocellular carcinoma (HCC) is the most prevalent primary malignancy of the liver and results in ~800,000 deaths globally per year (81). It is the fastest growing cancer in the US. Accumulation of tumor-associated macrophages is commonly seen in the livers of patients with HCC and the number of macrophages correlates with HCC progression and poor prognosis (82, 83). As key components of the tumor microenvironment, macrophages are thought to be pro-inflammatory and pro-tumorgenic during HCC progression, but may switch to become anti-tumorgenic during HCC regression (79). In zebrafish, HCC can be induced by carcinogen and mutagen treatment, genetic mutations of tumor suppressor genes and oncogenes, and transgenic overexpression of oncogenes (7, 84). Zebrafish and human HCCs share similar histological features and gene signatures (85, 86). Increases in macrophage numbers have been observed in zebrafish HCC models with different tumorigenic triggers (35, 61, 87, 88).

By live imaging, De Oliverira et al. showed that HFD feeding induces changes in macrophage morphology and polarization in a transgenic zebrafish HCC model expressing activated β-cateninin in the hepatocytes (61). Ablating macrophages prior to HFD feeding suppresses the exacerbated liver enlargement in HCC fish that is caused by HFD. Treatment with anti-diabetic agent metformin has similar inhibitory effects on HCC progression associated with HFD. Whereas, metformin has previously been proposed as a promising treatment for HCC, the zebrafish study provides direct *in vivo* evidence to show that it suppresses NAFLD-associated HCC progression by decreasing the number of pro-inflammatory macrophages and increasing T cell infiltration.

One challenge for investigating the roles of liver macrophages in HCC is that tumor formation often occurs in parallel with the progression of chronic liver disease. It is difficult to segregate the roles of macrophages in maintaining a prone-tumor inflammatory microenvironment vs. promoting HCC in response to tumor-derived signals (3). Multiple transgenic zebrafish lines utilizing chemically inducible expression systems (Tet-on, Tet-Off, and Mifepristone) have been generated to overexpress different oncogenes specifically in the hepatocytes (89). These models exclude the impact of chronic liver disease on HCC formation. Moreover, HCC can be induced in a temporally controlled manner and is regressed after removal of the chemicals, allowing investigation of macrophages at different stages of HCC progression and regression. In a transgenic zebrafish HCC model with inducible expression of oncogene *Xmrk* that encodes a hyperactive epidermal growth factor receptor (EGFR) homolog, the number of macrophages is increased during both HCC formation and regression (87). Interestingly, the macrophages are randomly distributed during HCC formation, and gradually show prominent blood vessel association as HCC regresses, implying that they have different functions at these two stages.

*In vivo* live imaging of the interactions between oncogenic hepatocytes and their microenvironment can be technically difficult in rodent models. Such analyses are readily feasible in zebrafish larvae due to their transparent body and availability of cell type-specific transgenic fluorescent reporter lines. Yang et al. investigated the responses of hepatocytes, innate immune cells and hepatic stellate cells during early stage of liver tumorigenesis in a *kras*^*v*12^-induced HCC model (63). Upon hepatocyte-specific *kras*^*v*12^ overexpression, there is sequential infiltration of neutrophils and macrophages, followed by proliferation and activation of hepatic stellate cells. Whereas, decreasing macrophage numbers by knocking down *irf8* or *pu.1* impairs both survival and activation of hepatic stellate cells, reducing neutrophils only affects their activation. The study further revealed reciprocal interactions between hepatic stellate cells and immune cells in HCC. Upon HCC induction, hepatocytes and macrophages increase expression levels of serotonin to regulate hepatic stellate cell survival and activation. In return, activated hepatic stellate cells secrete TGFβ1 to promote the pro-tumorigenesis function of neutrophils and macrophages. This work demonstrates the dynamic intercellular crosstalks within the tumor microenvironment that are crucial for liver tumorigenesis.

HCC is a male-biased disease with a male-to-female ratio of 2.4 worldwide (90). It is more aggressive and has worse prognosis in men than in women. The gender disparity also exists in rodent and zebrafish HCC models (91–93). In a series of reports from the Gong laboratory, the mechanisms of male-biased HCC carcinogenesis were explored in the transgenic zebrafish with inducible expression of oncogenes. In HCC models induced by *kras*^*v*12^ and *xmrk* expression, there is an enhancement of hepatocarcinogenesis in male zebrafish compared to females (35, 94). Male HCC livers express higher levels of serotonin. It is accompanied with higher numbers of total hepatic stellate cells and activated hepatic stellate cells, as well as more severe infiltration of macrophages and neutrophils. The sex disproportion of HCC is thought to be not only due to varying risk factors in men and women, but also associated with the regulation of inflammatory responses in the tumor microenvironment by sex hormones (95, 96). Yet, the results of estrogen- and androgen-related clinical trials are inconclusive (97–99), suggesting the possible involvement of other hormones. One candidate is cortisol that is predominantly expressed in the male livers (35, 100). In the zebrafish *kras*^*v*12^ and *xmrk* HCC models, cortisol induces expression of TGFβ1, which subsequently promotes infiltration of macrophages and neutrophils to accelerate hepatocarcinogenesis. The positive correlation between cortisol, TGFβ1, and macrophage/neutrophil infiltration has also been observed in patients with HCC (35).

## Macrophages in Zebrafish Models of Liver Regeneration

Aligned with their involvement in liver diseases, macrophages are key participants in liver regeneration (101–103). Upon injury, liver macrophages infiltrate to the wound site to remove the dead hepatocytes. They also produce cytokines IL6 and TNFα that prompt hepatocytes to enter the mitotic cycle. Depletion of Kupffer cells in rodents by clodronate liposomes delays liver regeneration and exaggerates liver damage after partial hepatectomy (104, 105). Three liver regeneration models have been characterized in depth in zebrafish and the contribution of macrophages has been investigated. Following one-third partial hepatectomy, the adult zebrafish liver regains its original volume within 14 days via compensatory growth of the remnant hepatocytes (106–108). Macrophages accumulate at the amputation site within 48 h after the surgery to clear up neutrophils and resolve local inflammation (109). Digestive-organ-expansion-factor (Def) is a nucleolar protein that mediates p53 degradation in the nucleolus. In zebrafish with haploinsufficiency of Def, aberrant expression of cytokines halts the timely migration of macrophages to the amputation site. The resulting delay in neutrophil clearance and prolonged inflammation cause fibrotic scar formation.

Two hepatocyte-specific ablation models have been established in zebrafish. In one model, the transgenic zebrafish expressing the oxygen-insensitive NAD(P)H nitroreducatse (NTR) in hepatocytes are treated with the antiprotozoal metronidazole. This prodrug is metabolized into a cytotoxin by NTR to induce rapid death of the hepatocytes (110, 111). Treatment with metronidazole from 3.5 to 5 days post fertilization results in nearly complete hepatocyte ablation. The liver size fully recovers just 5 days after removal of the drug (112). In a second model, temporary knockdown of mitochondrial importer gene *tomm22* by morpholino oligonucleotide leads to hepatocyte degeneration. The liver in the morpholino-injected animal is smaller at 4 days post fertilization, but starts to regenerate as the morpholino effect expires and *tomm22* expression is restored to the wild-type level (17). By 8 days post fertilization, the liver displays the size and structure that resemble the uninjected control. Unlike partial hepatectomy in which liver regeneration is driven by proliferation of existing hepatocytes, in both hepatocyte-NTR and *tomm22*-knockdown models, extensive hepatocyte loss triggers dedifferentiation of biliary epithelial cells into liver progenitor cells to form new hepatocytes (112, 113). Robust recruitment of macrophages and engulfment of hepatocyte debris by macrophages are seen in both models (17, 114). In *tomm22*-knockdown model, the surviving hepatocytes turn on biliary markers to become hybrid cells that express both hepatocyte and biliary markers (38). Ablation of macrophages suppresses the formation of hydrid cells, which coincides with the reduction of Wnt/β-catenin signaling activity. This is consistent with the mammalian findings that macrophages produce Wnt3a to promote liver progenitor cell differentiation toward the hepatocyte fate during regeneration (115).

By combining the liver regeneration models with the transgenic macrophage reporter lines, it is feasible to monitor macrophage recruitment, efferocytosis, and their interactions with other hepatic cells *in vivo* throughout the course of liver regeneration. The liver macrophages in *tomm22*-knockdown model exhibit a shift in morphology during the regeneration phase (38), suggesting that they undergo activation and polarization similar to their mammalian counterparts. In rodents, Kupffer cells and blood monocyte-derived macrophages play different roles in liver regeneration depending on the type of the original injury (2, 5). It will be interesting to utilize the zebrafish partial hepatectomy and hepatocyte depletion models to compare the source of macrophages and their functions in hepatocyte- and biliary-driven liver regeneration, respectively.

## Conclusion and Future Perspectives

Several possible strategies can be used to design macrophage-based treatment for acute and chronic liver diseases: (1) Suppressing Kupffer cell activation; (2) blocking monocyte recruitment; (3) rendering macrophages toward a more restorative phenotype; and (4) macrophage cell therapy (3, 116). Not every aspect of liver macrophage biology can be easily investigated using *in vitro* systems and rodent models and including complementary animal models will be beneficial. Zebrafish has the complexity of a vertebrate system, established models of acute and chronic liver injury, conserved innate immune cells, and superior genetic and live-imaging capabilities, making it an attractive alternative animal model for studying macrophages in liver homeostasis and diseases. In this review, we have discussed the strengths of using zebrafish to visualize macrophages and monitor their interactions with other hepatic cells, and to manipulate these cells using genetic approaches.

The characterization of zebrafish liver macrophages is only at the beginning stage and much remains to be learned. Transcriptomic analysis of zebrafish macrophages has been performed in the context of Mycobacterial infection (117). However, the macrophage transcriptome has not been investigated in healthy and injured liver in zebrafish and to what degree this is comparable to humans is not clear. Few macrophage-specific antibodies are available in zebrafish. In particular, cell surface markers labeling macrophages at different polarization states have yet to be identified. Most of the zebrafish liver studies are conducted on larvae, as live imaging becomes less feasible beyond the larval stage when they are no longer transparent. Fibrosis and cirrhosis, however, are chronic processes and the duration of the larvae studies may not be long enough for fibrosis to develop. Another caveat of studying larvae is that the zebrafish immune system is primarily innate during the first month of life and the adaptive system only becomes fully functional afterwards (50). On one hand, the temporal separation of innate and adaptive immune systems permits exclusive interrogation of innate immune cell function without having significant influence from adaptive immunity. On the other hand, pathogenesis of human liver diseases does involve both innate and adaptive immunity and it is necessary to validate the larval findings in adult liver disease models. Comparative studies on human vs. zebrafish liver macrophages in physiological and disease conditions are very limited, and thus the human relevance of zebrafish findings should be evaluated.

Liver macrophages are highly polymorphic. The lack of tools to distinguish macrophages from different origins and at different activation states has prevented the assignment of specific functionalities to each subgroup, making it difficult to develop treatment that only targets the macrophage subgroups with detrimental effect. Some emerging technologies in zebrafish may open exciting revenues for interrogating the ontogeny, activation, heterogeneity, and plasticity of liver macrophages. The zebrafish model possesses an excellent toolbox for lineage-tracing and fate-mapping analyses to understand the ontogeny of different liver macrophage subgroups in normal and diseased livers. For instance, the Zebrabow system allows tracing of the clonal origin of different liver macrophage subtypes (118, 119). Distinct clones can be sorted and sequenced separately to uncover the transcriptional states of different subpopulations. The multicolor labeling can also be utilized in adult zebrafish to assess the maintenance of liver macrophages population and distinguish between self-renewal and monocyte-based replenishment. It is possible to partially ablate the labeled macrophages by using clodronate liposomes (120). Subsequently, the clonal composition can be assessed to identify the source of the recovering cells. Moreover, the labeled clones can be analyzed to determine how different subpopulations of macrophages react to various insults. The GESTALT system, which stands for genome editing of synthetic target arrays for lineage tracing, is another tool to add more depth to the understanding of liver macrophage ontogeny (121). It utilizes CRISPR genome editing to progressively introduce and accumulate distinct mutations in a DNA barcode over multiple rounds of cell division. The barcode can be used to dissect lineage relationship among liver macrophages via the mutation patterns shared between them. With the use of a heat shock inducible Cas9, it is also possible to laser-activate the GESTALT system in a spatio-temporally restricted manner to restrict the labeling to a specific site of hematopoiesis and study the lineage relationship within this particular group. The GESTALT method can be combined with single-cell RNA sequencing to not only provide the identity of the subpopulations but also link each of them to a specific hematopoietic lineage and site (122). One may apply the GESTALT method in different liver pathologies to evaluate the liver macrophages plasticity at a population level. Lastly, a recent study from Paul and colleagues describes successful transplantation of primary human monocytes/macrophages into larval zebrafish, both directly into circulation and in an organ-specific manner (123). The human monocytes differentiate into functional macrophages at the physiological temperature of zebrafish, and survive for at least 2weeks in the presence of zebrafish immunity. This methodology may permit *in vivo* characterization of human macrophages in zebrafish models of liver pathology at a cellular level. The new lines of experiments described above have the potential to advance our understanding of liver macrophage biology and contribute to the design of novel macrophage-targeted therapeutic strategies to treat liver diseases.

## Author Contributions

All authors listed have made a substantial, direct and intellectual contribution to the work, and approved it for publication.

### Conflict of Interest

The authors declare that the research was conducted in the absence of any commercial or financial relationships that could be construed as a potential conflict of interest.

## References

[1] Asrani SK Devarbhavi H Eaton J Kamath PS. Burden of liver diseases in the world. J Hepatol. (2019) 70:151–71. 10.1016/j.jhep.2018.09.014 30266282

[2] Elchaninov AV Fatkhudinov TK Vishnyakova PA Lokhonina AV Sukhikh GT. Phenotypical and functional polymorphism of liver resident macrophages. Cells. (2019) 8:E1032. 10.3390/cells8091032 31491903 PMC6769646

[3] Tacke F. Targeting hepatic macrophages to treat liver diseases. J Hepatol. (2017) 66:1300–12. 10.1016/j.jhep.2017.02.026 28267621

[4] Ramachandran P Dobie R Wilson-Kanamori JR Dora EF Henderson BEP Luu NT. Resolving the fibrotic niche of human liver cirrhosis at single-cell level. Nature. (2019) 575:512–8. 10.1101/766113 31597160 PMC6876711

[5] Guillot A Tacke F. Liver macrophages: old dogmas and new insights. Hepatol Commun. (2019) 3:730–43. 10.1002/hep4.1356 31168508 PMC6545867

[6] Pham DH Zhang C Yin C. Using zebrafish to model liver diseases-Where do we stand? Curr Pathobiol Rep. (2017) 5:207–21. 10.1007/s40139-017-0141-y 29098121 PMC5662119

[7] Goessling W Sadler KC. Zebrafish: an important tool for liver disease research. Gastroenterology. (2015) 149:1361–77. 10.1053/j.gastro.2015.08.034 26319012 PMC4762709

[8] Wang S Miller SR Ober EA Sadler KC. Making It new again: insight into liver development, regeneration, and disease from zebrafish research. Curr Top Dev Biol. (2017) 124:161–95. 10.1016/bs.ctdb.2016.11.012 28335859 PMC6450094

[9] Astin JW Keerthisinghe P Du L Sanderson LE Crosier KE Crosier PS. Innate immune cells and bacterial infection in zebrafish. Methods Cell Biol. (2017) 138:31–60. 10.1016/bs.mcb.2016.08.002 28129850

[10] Herbomel P Thisse B Thisse C. Zebrafish early macrophages colonize cephalic mesenchyme and developing brain, retina, and epidermis through a M-CSF receptor-dependent invasive process. Dev Biol. (2001) 238:274–88. 10.1006/dbio.2001.0393 11784010

[11] Parichy DM Ransom DG Paw B Zon LI Johnson SL. An orthologue of the kit-related gene fms is required for development of neural crest-derived xanthophores and a subpopulation of adult melanocytes in the zebrafish, Danio rerio. Development. (2000) 127:3031–44. 10862741 10.1242/dev.127.14.3031

[12] Zakrzewska A Cui C Stockhammer OW Benard EL Spaink HP Meijer AH. Macrophage-specific gene functions in Spi1-directed innate immunity. Blood. (2010) 116:e1–11. 10.1182/blood-2010-01-262873 20424185

[13] Ellett F Pase L Hayman JW Andrianopoulos A Lieschke GJ. mpeg1 promoter transgenes direct macrophage-lineage expression in zebrafish. Blood. (2011) 117:e49–56. 10.1182/blood-2010-10-314120 21084707 PMC3056479

[14] Benard EL Racz PI Rougeot J Nezhinsky AE Verbeek FJ Spaink HP. Macrophage-expressed perforins mpeg1 and mpeg1.2 have an anti-bacterial function in zebrafish. J Innate Immun. (2015) 7:136–52. 10.1159/000366103 25247677 PMC6738794

[15] Martins RR Ellis PS MacDonald RB Richardson RJ Henriques CM. Resident immunity in tissue repair and maintenance: the Zebrafish model coming of age. Front Cell Dev Biol. (2019) 7:12. 10.3389/fcell.2019.00012 30805338 PMC6370978

[16] Redd MJ Kelly G Dunn G Way M Martin P. Imaging macrophage chemotaxis in vivo: studies of microtubule function in zebrafish wound inflammation. Cell Motil Cytoskeleton. (2006) 63:415–22. 10.1002/cm.20133 16671106

[17] Curado S Ober EA Walsh S Cortes-Hernandez P Verkade H Koehler CM. The mitochondrial import gene tomm22 is specifically required for hepatocyte survival and provides a liver regeneration model. Dis Model Mech. (2010) 3:486–95. 10.1242/dmm.004390 20483998 PMC2898538

[18] van der Sar AM Appelmelk BJ Vandenbroucke-Grauls CM Bitter W. A star with stripes: zebrafish as an infection model. Trends Microbiol. (2004) 12:451–7. 10.1016/j.tim.2004.08.001 15381194

[19] Harvie EA Green JM Neely MN Huttenlocher A. Innate immune response to Streptococcus iniae infection in zebrafish larvae. Infect Immun. (2013) 81:110–21. 10.1128/IAI.00642-12 23090960 PMC3536132

[20] Wu S Xue R Hassan S Nguyen TML Wang T Pan H. Il34-Csf1r pathway regulates the migration and colonization of microglial precursors. Dev Cell. (2018) 46:552–63 e4. 10.1016/j.devcel.2018.08.005 30205037

[21] Roh-Johnson M Shah AN Stonick JA Poudel KR Kargl J Yang GH. Macrophage-dependent cytoplasmic transfer during melanoma invasion *in vivo*. Dev Cell. (2017) 43:549–62 e6. 10.1016/j.devcel.2017.11.003 29207258 PMC5728704

[22] Gray C Loynes CA Whyte MK Crossman DC Renshaw SA Chico TJ. Simultaneous intravital imaging of macrophage and neutrophil behaviour during inflammation using a novel transgenic zebrafish. Thromb Haemost. (2011) 105:811–9. 10.1160/TH10-08-0525 21225092

[23] Dee CT Nagaraju RT Athanasiadis EI Gray C Fernandez Del Ama L Johnston SA. CD4-transgenic Zebrafish reveal tissue-resident Th2- and regulatory T cell-like populations and diverse mononuclear phagocytes. J Immunol. (2016) 197:3520–30. 10.4049/jimmunol.1600959 27694495 PMC5073357

[24] Oehlers SH Cronan MR Scott NR Thomas MI Okuda KS Walton EM. Interception of host angiogenic signalling limits mycobacterial growth. Nature. (2015) 517:612–5. 10.1038/nature13967 25470057 PMC4312197

[25] Walton EM Cronan MR Beerman RW Tobin DM. The Macrophage-specific promoter mfap4 allows live, long-term analysis of macrophage behavior during mycobacterial infection in Zebrafish. PLoS ONE. (2015) 10:e0138949. 10.1371/journal.pone.0138949 26445458 PMC4596833

[26] Nguyen-Chi M Laplace-Builhe B Travnickova J Luz-Crawford P Tejedor G Phan QT. Identification of polarized macrophage subsets in zebrafish. Elife. (2015) 4:e07288. 10.7554/eLife.07288 26154973 PMC4521581

[27] Sanderson LE Chien AT Astin JW Crosier KE Crosier PS Hall CJ. An inducible transgene reports activation of macrophages in live zebrafish larvae. Dev Comp Immunol. (2015) 53:63–9. 10.1016/j.dci.2015.06.013 26123890

[28] Li L Yan B Shi YQ Zhang WQ Wen ZL. Live imaging reveals differing roles of macrophages and neutrophils during zebrafish tail fin regeneration. J Biol Chem. (2012) 287:25353–60. 10.1074/jbc.M112.349126 22573321 PMC3408142

[29] Van Rooijen N Sanders A. Liposome mediated depletion of macrophages: mechanism of action, preparation of liposomes and applications. J Immunol Methods. (1994) 174:83–93. 10.1016/0022-1759(94)90012-4 8083541

[30] Bernut A Herrmann JL Kissa K Dubremetz JF Gaillard JL Lutfalla G. *Mycobacterium abscessus* cording prevents phagocytosis and promotes abscess formation. Proc Natl Acad Sci USA. (2014) 111:E943–52. 10.1073/pnas.1321390111 24567393 PMC3956181

[31] Phelps HA Neely MN. SalY of the Streptococcus pyogenes lantibiotic locus is required for full virulence and intracellular survival in macrophages. Infect Immun. (2007) 75:4541–51. 10.1128/IAI.00518-07 17576754 PMC1951192

[32] Chatani M Takano Y Kudo A. Osteoclasts in bone modeling, as revealed by in vivo imaging, are essential for organogenesis in fish. Dev Biol. (2011) 360:96–109. 10.1016/j.ydbio.2011.09.013 21963458

[33] Yan C Huo X Wang S Feng Y Gong Z. Stimulation of hepatocarcinogenesis by neutrophils upon induction of oncogenic kras expression in transgenic zebrafish. J Hepatol. (2015) 63:420–8. 10.1016/j.jhep.2015.03.024 25828472 PMC4508360

[34] Shiau CE Kaufman Z Meireles AM Talbot WS. Differential requirement for irf8 in formation of embryonic and adult macrophages in zebrafish. PLoS ONE. (2015) 10:e0117513. 10.1371/journal.pone.0117513 25615614 PMC4304715

[35] Yan C Yang Q Gong Z. Tumor-associated neutrophils and macrophages promote gender disparity in hepatocellular carcinoma in Zebrafish. Cancer Res. (2017) 77:1395–407. 10.1158/0008-5472.CAN-16-2200 28202512

[36] Su F Juarez MA Cooke CL Lapointe L Shavit JA Yamaoka JS. Differential regulation of primitive myelopoiesis in the zebrafish by Spi-1/Pu.1 and C/ebp1. Zebrafish. (2007) 4:187–99. 10.1089/zeb.2007.0505 18041923

[37] Petrie TA Strand NS Yang CT Rabinowitz JS Moon RT. Macrophages modulate adult zebrafish tail fin regeneration. Development. (2014) 141:2581–91. 10.1242/dev.098459 24961798 PMC4067955

[38] Wu J Choi TY Shin D. tomm22 knockdown-mediated hepatocyte damages elicit both the formation of hybrid hepatocytes and biliary conversion to hepatocytes in Zebrafish Larvae. Gene Expr. (2017) 17:237–49. 10.3727/105221617X695195 28251883 PMC5542045

[39] Torraca V Cui C Boland R Bebelman JP van der Sar AM Smit MJ. The CXCR3-CXCL11 signaling axis mediates macrophage recruitment and dissemination of mycobacterial infection. Dis Model Mech. (2015) 8:253–69. 10.1242/dmm.017756 25573892 PMC4348563

[40] Evans MA Smart N Dube KN Bollini S Clark JE Evans HG. Thymosin beta4-sulfoxide attenuates inflammatory cell infiltration and promotes cardiac wound healing. Nat Commun. (2013) 4:2081. 10.1038/ncomms3081 23820300 PMC3797509

[41] Jagannathan-Bogdan M Zon LI. Hematopoiesis. Development. (2013) 140:2463–7. 10.1242/dev.083147 23715539 PMC3666375

[42] Stachura DL Traver D. Cellular dissection of zebrafish hematopoiesis. Methods Cell Biol. (2011) 101:75–110. 10.1016/B978-0-12-387036-0.00004-9 21550440

[43] Bertrand JY Kim AD Violette EP Stachura DL Cisson JL Traver D. Definitive hematopoiesis initiates through a committed erythromyeloid progenitor in the zebrafish embryo. Development. (2007) 134:4147–56. 10.1242/dev.012385 17959717 PMC2735398

[44] Goessling W North TE. Hematopoietic stem cell development: using the zebrafish to identify the signaling networks and physical forces regulating hematopoiesis. Methods Cell Biol. (2011) 105:117–36. 10.1016/B978-0-12-381320-6.00005-9 21951528

[45] Orkin SH Zon LI. Hematopoiesis: an evolving paradigm for stem cell biology. Cell. (2008) 132:631–44. 10.1016/j.cell.2008.01.025 18295580 PMC2628169

[46] Murayama E Kissa K Zapata A Mordelet E Briolat V Lin HF. Tracing hematopoietic precursor migration to successive hematopoietic organs during zebrafish development. Immunity. (2006) 25:963–75. 10.1016/j.immuni.2006.10.015 17157041

[47] Bertrand JY Kim AD Teng S Traver D. CD41+ cmyb+ precursors colonize the zebrafish pronephros by a novel migration route to initiate adult hematopoiesis. Development. (2008) 135:1853–62. 10.1242/dev.015297 18417622 PMC2762343

[48] Bennett CM Kanki JP Rhodes J Liu TX Paw BH Kieran MW. Myelopoiesis in the zebrafish, Danio rerio. Blood. (2001) 98:643–51. 10.1182/blood.V98.3.643 11468162

[49] Hsu K Traver D Kutok JL Hagen A Liu TX Paw BH. The pu.1 promoter drives myeloid gene expression in zebrafish. Blood. (2004) 104:1291–7. 10.1182/blood-2003-09-3105 14996705

[50] Lam SH Chua HL Gong Z Lam TJ Sin YM. Development and maturation of the immune system in zebrafish, Danio rerio: a gene expression profiling, in situ hybridization and immunological study. Dev Comp Immunol. (2004) 28:9–28. 10.1016/S0145-305X(03)00103-4 12962979

[51] Varol C Mildner A Jung S. Macrophages: development and tissue specialization. Annu Rev Immunol. (2015) 33:643–75. 10.1146/annurev-immunol-032414-112220 25861979

[52] Ginhoux F Guilliams M. Tissue-resident macrophage ontogeny and homeostasis. Immunity. (2016) 44:439–49. 10.1016/j.immuni.2016.02.024 26982352

[53] Hoeffel G Ginhoux F. Ontogeny of tissue-resident macrophages. Front Immunol. (2015) 6:486. 10.3389/fimmu.2015.00486 26441990 PMC4585135

[54] Perdiguero EG Klapproth K Schulz C Busch K de Bruijn M Rodewald HR. The origin of tissue-resident macrophages: when an erythro-myeloid progenitor is an erythro-myeloid progenitor. Immunity. (2015) 43:1023–4. 10.1016/j.immuni.2015.11.022 26682973

[55] Schulz C Gomez Perdiguero E Chorro L Szabo-Rogers H Cagnard N Kierdorf K. A lineage of myeloid cells independent of Myb and hematopoietic stem cells. Science. (2012) 336:86–90. 10.1126/science.1219179 22442384

[56] Bain CC Bravo-Blas A Scott CL Perdiguero EG Geissmann F Henri S. Constant replenishment from circulating monocytes maintains the macrophage pool in the intestine of adult mice. Nat Immunol. (2014) 15:929–37. 10.1038/ni.2967 25151491 PMC4169290

[57] Ferrero G Mahony CB Dupuis E Yvernogeau L Di Ruggiero E Miserocchi M. Embryonic microglia derive from primitive macrophages and are replaced by cmyb-dependent definitive microglia in Zebrafish. Cell Rep. (2018) 24:130–41. 10.1016/j.celrep.2018.05.066 29972775

[58] He S Chen J Jiang Y Wu Y Zhu L Jin W. Adult zebrafish Langerhans cells arise from hematopoietic stem/progenitor cells. Elife. (2018) 7:e36131. 10.7554/eLife.36131 29905527 PMC6017808

[59] Sheng J Ruedl C Karjalainen K. Most Tissue-resident macrophages except microglia are derived from fetal hematopoietic stem cells. Immunity. (2015) 43:382–93. 10.1016/j.immuni.2015.07.016 26287683

[60] Mass E Ballesteros I Farlik M Halbritter F Gunther P Crozet L. Specification of tissue-resident macrophages during organogenesis. Science. (2016) 353:aaf4238. 10.1126/science.aaf4238 27492475 PMC5066309

[61] de Oliveira S Houseright RA Graves AL Golenberg N Korte BG Miskolci V. Metformin modulates innate immune-mediated inflammation and early progression of NAFLD-associated hepatocellular carcinoma in zebrafish. J Hepatol. (2019) 70:710–21. 10.1016/j.jhep.2018.11.034 30572006 PMC6436385

[62] Wittamer V Bertrand JY Gutschow PW Traver D. Characterization of the mononuclear phagocyte system in zebrafish. Blood. (2011) 117:7126–35. 10.1182/blood-2010-11-321448 21406720

[63] Yang Q Yan C Gong Z. Interaction of hepatic stellate cells with neutrophils and macrophages in the liver following oncogenic kras activation in transgenic zebrafish. Sci Rep. (2018) 8:8495. 10.1038/s41598-018-26612-0 29855567 PMC5981472

[64] Vonghia L Van Herck MA Weyler J Francque S. Targeting myeloid-derived cells: new frontiers in the treatment of non-alcoholic and alcoholic liver disease. Front Immunol. (2019) 10:563. 10.3389/fimmu.2019.00563 30972062 PMC6446913

[65] Bartneck M Fech V Ehling J Govaere O Warzecha KT Hittatiya K. Histidine-rich glycoprotein promotes macrophage activation and inflammation in chronic liver disease. Hepatology. (2016) 63:1310–24. 10.1002/hep.28418 26699087

[66] Wan J Benkdane M Teixeira-Clerc F Bonnafous S Louvet A Lafdil F. M2 Kupffer cells promote M1 Kupffer cell apoptosis: a protective mechanism against alcoholic and nonalcoholic fatty liver disease. Hepatology. (2014) 59:130–42. 10.1002/hep.26607 23832548

[67] Wan J Benkdane M Alons E Lotersztajn S Pavoine C. M2 kupffer cells promote hepatocyte senescence: an IL-6-dependent protective mechanism against alcoholic liver disease. Am J Pathol. (2014) 184:1763–72. 10.1016/j.ajpath.2014.02.014 24713392

[68] Ma J Yin H Li M Deng Y Ahmad O Qin G. A Comprehensive study of high cholesterol diet-induced larval zebrafish model: a short-time *in vivo* screening method for non-alcoholic fatty liver disease drugs. Int J Biol Sci. (2019) 15:973–83. 10.7150/ijbs.30013 31182918 PMC6535789

[69] Lin JN Chang LL Lai CH Lin KJ Lin MF Yang CH. Development of an animal model for alcoholic liver disease in Zebrafish. Zebrafish. (2015) 12:271–80. 10.1089/zeb.2014.1054 25923904

[70] Park KH Kim SH. Low dose of chronic ethanol exposure in adult zebrafish induces hepatic steatosis and injury. Biomed Pharmacother. (2019) 117:109179. 10.1016/j.biopha.2019.109179 31387182 PMC6686888

[71] Passeri MJ Cinaroglu A Gao C Sadler KC. Hepatic steatosis in response to acute alcohol exposure in zebrafish requires sterol regulatory element binding protein activation. Hepatology. (2009) 49:443–52. 10.1002/hep.22667 19127516 PMC2635426

[72] Schneider AC Gregorio C Uribe-Cruz C Guizzo R Malysz T Faccioni-Heuser MC. Chronic exposure to ethanol causes steatosis and inflammation in zebrafish liver. World J Hepatol. (2017) 9:418–26. 10.4254/wjh.v9.i8.418 28357029 PMC5355764

[73] Zhang C Ellis JL Yin C. Inhibition of vascular endothelial growth factor signaling facilitates liver repair from acute ethanol-induced injury in zebrafish. Dis Model Mech. (2016) 9:1383–96. 10.1242/dmm.024950 27562099 PMC5117223

[74] Cederbaum AI. Alcohol metabolism. Clin Liver Dis. (2012) 16:667–85. 10.1016/j.cld.2012.08.002 23101976 PMC3484320

[75] Tran S Nowicki M Chatterjee D Gerlai R. Acute and chronic ethanol exposure differentially alters alcohol dehydrogenase and aldehyde dehydrogenase activity in the zebrafish liver. Prog Neuropsychopharmacol Biol Psychiatry. (2015) 56:221–6. 10.1016/j.pnpbp.2014.09.011 25290637

[76] Tsedensodnom O Vacaru AM Howarth DL Yin C Sadler KC. Ethanol metabolism and oxidative stress are required for unfolded protein response activation and steatosis in zebrafish with alcoholic liver disease. Dis Model Mech. (2013) 6:1213–26. 10.1242/dmm.012195 23798569 PMC3759341

[77] Dunn W Shah VH. Pathogenesis of alcoholic liver disease. Clin Liver Dis. (2016) 20:445–56. 10.1016/j.cld.2016.02.004 27373608 PMC4933837

[78] Howarth DL Passeri M Sadler KC. Drinks like a fish: using zebrafish to understand alcoholic liver disease. Alcohol Clin Exp Res. (2011) 35:826–9. 10.1111/j.1530-0277.2010.01407.x 21284674 PMC3083479

[79] Krenkel O Tacke F. Liver macrophages in tissue homeostasis and disease. Nat Rev Immunol. (2017) 17:306–21. 10.1038/nri.2017.11 28317925

[80] Duffield JS Forbes SJ Constandinou CM Clay S Partolina M Vuthoori S. Selective depletion of macrophages reveals distinct, opposing roles during liver injury and repair. J Clin Invest. (2005) 115:56–65. 10.1172/JCI22675 15630444 PMC539199

[81] Global Burden of Disease Liver Cancer C Akinyemiju T Abera S Ahmed M Alam N Alemayohu MA. The burden of primary liver cancer and underlying etiologies from 1990 to 2015 at the global, regional, and national level: results from the global burden of disease study 2015. JAMA Oncol. (2017) 3:1683–91. 10.1001/jamaoncol.2017.3055 28983565 PMC5824275

[82] Ding T Xu J Wang F Shi M Zhang Y Li SP. High tumor-infiltrating macrophage density predicts poor prognosis in patients with primary hepatocellular carcinoma after resection. Hum Pathol. (2009) 40:381–9. 10.1016/j.humpath.2008.08.011 18992916

[83] Yeung OW Lo CM Ling CC Qi X Geng W Li CX. Alternatively activated (M2) macrophages promote tumour growth and invasiveness in hepatocellular carcinoma. J Hepatol. (2015) 62:607–16. 10.1016/j.jhep.2014.10.029 25450711

[84] Wrighton PJ Oderberg IM Goessling W. There is something fishy about liver cancer: zebrafish models of hepatocellular carcinoma. Cell Mol Gastroenterol Hepatol. (2019) 8:347–63. 10.1016/j.jcmgh.2019.05.002 31108233 PMC6713889

[85] Lam SH Wu YL Vega VB Miller LD Spitsbergen J Tong Y. Conservation of gene expression signatures between zebrafish and human liver tumors and tumor progression. Nat Biotechnol. (2006) 24:73–5. 10.1038/nbt1169 16327811

[86] Zheng W Li Z Nguyen AT Li C Emelyanov A Gong Z. Xmrk, kras and myc transgenic zebrafish liver cancer models share molecular signatures with subsets of human hepatocellular carcinoma. PLoS ONE. (2014) 9:e91179. 10.1371/journal.pone.0091179 24633177 PMC3954698

[87] Li Z Luo H Li C Huo X Yan C Huang X. Transcriptomic analysis of a transgenic zebrafish hepatocellular carcinoma model reveals a prominent role of immune responses in tumour progression and regression. Int J Cancer. (2014) 135:1564–73. 10.1002/ijc.28794 24550086

[88] Yan C Yang Q Gong Z. Transgenic expression of tgfb1a induces hepatic inflammation, fibrosis and metastasis in zebrafish. Biochem Biophys Res Commun. (2019) 509:175–81. 10.1016/j.bbrc.2018.12.098 30581008

[89] Lu JW Ho YJ Yang YJ Liao HA Ciou SC Lin LI. Zebrafish as a disease model for studying human hepatocellular carcinoma. World J Gastroenterol. (2015) 21:12042–58. 10.3748/wjg.v21.i42.12042 26576090 PMC4641123

[90] Parkin DM Bray F Ferlay J Pisani P. Global cancer statistics, 2002. CA Cancer J Clin. (2005) 55:74–108. 10.3322/canjclin.55.2.74 15761078

[91] Li H Lu JW Huo X Li Y Li Z Gong Z. Effects of sex hormones on liver tumor progression and regression in Myc/xmrk double oncogene transgenic zebrafish. Gen Comp Endocrinol. (2019) 277:112–21. 10.1016/j.ygcen.2019.03.018 30926469

[92] Naugler WE Sakurai T Kim S Maeda S Kim K Elsharkawy AM. Gender disparity in liver cancer due to sex differences in MyD88-dependent IL-6 production. Science. (2007) 317:121–4. 10.1126/science.1140485 17615358

[93] Wolf MJ Adili A Piotrowitz K Abdullah Z Boege Y Stemmer K. Metabolic activation of intrahepatic CD8+ T cells and NKT cells causes nonalcoholic steatohepatitis and liver cancer via cross-talk with hepatocytes. Cancer Cell. (2014) 26:549–64. 10.1016/j.ccell.2014.09.003 25314080

[94] Yang Q Yan C Gong Z. Activation of liver stromal cells is associated with male-biased liver tumor initiation in xmrk and Myc transgenic zebrafish. Sci Rep. (2017) 7:10315. 10.1038/s41598-017-10529-1 28871112 PMC5583234

[95] Iyer JK Kalra M Kaul A Payton ME Kaul R. Estrogen receptor expression in chronic hepatitis C and hepatocellular carcinoma pathogenesis. World J Gastroenterol. (2017) 23:6802–16. 10.3748/wjg.v23.i37.6802 29085224 PMC5645614

[96] Ma WL Lai HC Yeh S Cai X Chang C. Androgen receptor roles in hepatocellular carcinoma, fatty liver, cirrhosis and hepatitis. Endocr Relat Cancer. (2014) 21:R165–82. 10.1530/ERC-13-0283 24424503 PMC4165608

[97] Chow PK Machin D Chen Y Zhang X Win KM Hoang HH. Randomised double-blind trial of megestrol acetate vs placebo in treatment-naive advanced hepatocellular carcinoma. Br J Cancer. (2011) 105:945–52. 10.1038/bjc.2011.333 21863030 PMC3185948

[98] Chow PK Tai BC Tan CK Machin D Win KM Johnson PJ. High-dose tamoxifen in the treatment of inoperable hepatocellular carcinoma: a multicenter randomized controlled trial. Hepatology. (2002) 36:1221–6. 10.1053/jhep.2002.36824 12395333

[99] Manesis EK Giannoulis G Zoumboulis P Vafiadou I Hadziyannis SJ. Treatment of hepatocellular carcinoma with combined suppression and inhibition of sex hormones: a randomized, controlled trial. Hepatology. (1995) 21:1535–42. 10.1002/hep.1840210610 7768497

[100] Van Cauter E Leproult R Kupfer DJ. Effects of gender and age on the levels and circadian rhythmicity of plasma cortisol. J Clin Endocrinol Metab. (1996) 81:2468–73. 10.1210/jc.81.7.2468 8675562

[101] Cressman DE Greenbaum LE DeAngelis RA Ciliberto G Furth EE Poli V. Liver failure and defective hepatocyte regeneration in interleukin-6-deficient mice. Science. (1996) 274:1379–83. 10.1126/science.274.5291.1379 8910279

[102] Michalopoulos GK. Liver regeneration after partial hepatectomy: critical analysis of mechanistic dilemmas. Am J Pathol. (2010) 176:2–13. 10.2353/ajpath.2010.090675 20019184 PMC2797862

[103] Webber EM Bruix J Pierce RH Fausto N. Tumor necrosis factor primes hepatocytes for DNA replication in the rat. Hepatology. (1998) 28:1226–34. 10.1002/hep.510280509 9794905

[104] Abshagen K Eipel C Kalff JC Menger MD Vollmar B. Loss of NF-kappaB activation in Kupffer cell-depleted mice impairs liver regeneration after partial hepatectomy. Am J Physiol Gastrointest Liver Physiol. (2007) 292:G1570–7. 10.1152/ajpgi.00399.2006 17322066

[105] Meijer C Wiezer MJ Diehl AM Schouten HJ Schouten HJ Meijer S. Kupffer cell depletion by CI2MDP-liposomes alters hepatic cytokine expression and delays liver regeneration after partial hepatectomy. Liver. (2000) 20:66–77. 10.1034/j.1600-0676.2000.020001066.x 10726963

[106] Goessling W North TE Lord AM Ceol C Lee S Weidinger G. APC mutant zebrafish uncover a changing temporal requirement for wnt signaling in liver development. Dev Biol. (2008) 320:161–74. 10.1016/j.ydbio.2008.05.526 18585699

[107] Kan NG Junghans D Izpisua Belmonte JC. Compensatory growth mechanisms regulated by BMP and FGF signaling mediate liver regeneration in zebrafish after partial hepatectomy. FASEB J. (2009) 23:3516–25. 10.1096/fj.09-131730 19546304 PMC2747679

[108] Sadler KC Krahn KN Gaur NA Ukomadu C. Liver growth in the embryo and during liver regeneration in zebrafish requires the cell cycle regulator, uhrf1. Proc Natl Acad Sci USA. (2007) 104:1570–5. 10.1073/pnas.0610774104 17242348 PMC1785278

[109] Zhu Z Chen J Xiong JW Peng J. Haploinsufficiency of Def activates p53-dependent TGFbeta signalling and causes scar formation after partial hepatectomy. PLoS ONE. (2014) 9:e96576. 10.1371/journal.pone.0096576 24801718 PMC4011785

[110] Curado S Anderson RM Jungblut B Mumm J Schroeter E Stainier DY. Conditional targeted cell ablation in zebrafish: a new tool for regeneration studies. Dev Dyn. (2007) 236:1025–35. 10.1002/dvdy.21100 17326133

[111] Curado S Stainier DY Anderson RM. Nitroreductase-mediated cell/tissue ablation in zebrafish: a spatially and temporally controlled ablation method with applications in developmental and regeneration studies. Nat Protoc. (2008) 3:948–54. 10.1038/nprot.2008.58 18536643 PMC2705989

[112] Choi TY Ninov N Stainier DY Shin D. Extensive conversion of hepatic biliary epithelial cells to hepatocytes after near total loss of hepatocytes in zebrafish. Gastroenterology. (2014) 146:776–88. 10.1053/j.gastro.2013.10.019 24148620 PMC3943869

[113] He J Lu H Zou Q Luo L. Regeneration of liver after extreme hepatocyte loss occurs mainly via biliary transdifferentiation in zebrafish. Gastroenterology. (2014) 146:789–800 e8. 10.1053/j.gastro.2013.11.045 24315993

[114] Stoddard M Huang C Enyedi B Niethammer P. Live imaging of leukocyte recruitment in a zebrafish model of chemical liver injury. Sci Rep. (2019) 9:28. 10.1038/s41598-018-36771-9 30631093 PMC6328554

[115] Boulter L Govaere O Bird TG Radulescu S Ramachandran P Pellicoro A. Macrophage-derived Wnt opposes Notch signaling to specify hepatic progenitor cell fate in chronic liver disease. Nat Med. (2012) 18:572–9. 10.1038/nm.2667 22388089 PMC3364717

[116] Starkey Lewis PJ Moroni F Forbes SJ. Macrophages as a cell-based therapy for liver disease. Semin Liver Dis. (2019) 39:442–51. 10.1055/s-0039-1688502 31242527

[117] Rougeot J Torraca V Zakrzewska A Kanwal Z Jansen HJ Sommer F. RNAseq profiling of leukocyte populations in zebrafish larvae reveals a cxcl11 chemokine gene as a marker of macrophage polarization during mycobacterial infection. Front Immunol. (2019) 10:832. 10.3389/fimmu.2019.00832 31110502 PMC6499218

[118] Henninger J Santoso B Hans S Durand E Moore J Mosimann C Clonal fate mapping quantifies the number of haematopoietic stem cells that arise during development. Nat Cell Biol. (2017) 19:17–27. 10.1038/ncb3444 28139650 PMC5898623

[119] Pan YA Freundlich T Weissman TA Schoppik D Wang XC Zimmerman S. Zebrabow: multispectral cell labeling for cell tracing and lineage analysis in zebrafish. Development. (2013) 140:2835–46. 10.1242/dev.094631 23757414 PMC3678346

[120] Wu Z Koh B Lawrence LM Kanamala M Pool B Svirskis D. Liposome-mediated drug delivery in larval Zebrafish to manipulate macrophage function. Zebrafish. (2019) 16:171–81. 10.1089/zeb.2018.1681 30724716

[121] McKenna A Findlay GM Gagnon JA Horwitz MS Schier AF Shendure J. Whole-organism lineage tracing by combinatorial and cumulative genome editing. Science. (2016) 353:aaf7907. 10.1126/science.aaf7907 27229144 PMC4967023

[122] Raj B Wagner DE McKenna A Pandey S Klein AM Shendure J. Simultaneous single-cell profiling of lineages and cell types in the vertebrate brain. Nat Biotechnol. (2018) 36:442–50. 10.1038/nbt.4103 29608178 PMC5938111

[123] Paul CD Devine A Bishop K Xu Q Wulftange WJ Burr H. Human macrophages survive and adopt activated genotypes in living zebrafish. Sci Rep. (2019) 9:1759. 10.1038/s41598-018-38186-y 30741975 PMC6370805

